# Kidneys Under Siege: Pesticides Impact Renal Health in the Freshwater Fish Common Carp (*Cyprinus carpio* Linnaeus, 1758)

**DOI:** 10.3390/toxics13070518

**Published:** 2025-06-20

**Authors:** Stela Stoyanova, Elenka Georgieva, Eleonora Kovacheva, László Antal, Dóra Somogyi, Ifeanyi Emmanuel Uzochukwu, László Nagy, Krisztián Nyeste, Vesela Yancheva

**Affiliations:** 1 Department of Developmental Biology, Paisii Hilendarski University of Plovdiv, 4000 Plovdiv, Bulgaria; stela.stoyanova@uni-plovdiv.bg (S.S.); elenkageorgieva@uni-plovdiv.bg (E.G.); 2 Department of Medical Biology, Medical University-Plovdiv, 4000 Plovdiv, Bulgaria; eleonora.t.p95@gmail.com; 3 Research Institute, Medical University-Plovdiv, 4000 Plovdiv, Bulgaria; 4 Department of Hydrobiology, University of Debrecen, 4032 Debrecen, Hungary; antal.laszlo@science.unideb.hu (L.A.); somogyi.dora@science.unideb.hu (D.S.); ifeanyi.uzochukwu@science.unideb.hu (I.E.U.); nagy.laszlo@science.unideb.hu (L.N.); 5 National Laboratory for Water Science and Water Safety, University of Debrecen, 4032 Debrecen, Hungary; 6 Pál Juhász-Nagy Doctoral School of Biology and Environmental Sciences, University of Debrecen, 4032 Debrecen, Hungary; 7 Department of Ecology and Environmental Conservation, Paisii Hilendarski University of Plovdiv, 4000 Plovdiv, Bulgaria; vyancheva@uni-plovdiv.bg

**Keywords:** common carp, kidney, histological biomarkers, pesticides, pollution

## Abstract

This study evaluated the histopathological impact of three commonly used pesticides—pirimiphos-methyl, propamocarb hydrochloride, and 2,4-dichlorophenoxyacetic acid (2,4-D)—on the kidneys of common carp (*Cyprinus carpio* Linnaeus, 1758) after 96-h acute exposure. The histopathological analysis demonstrated that all three tested pesticides induced structural changes. The histopathological changes were assessed using a semi-quantitative scoring system and categorised into circulatory, degenerative, proliferative, and inflammatory alterations. While circulatory alterations were absent in all treatments, clear and statistically significant degenerative, proliferative, and inflammatory responses were recorded, which escalated with increasing pesticide concentrations. Additionally, various statistical analyses were conducted to evaluate the lesions in kidney structure and function. Before the statistical analysis, normality and variance homogeneity were assessed using the Shapiro–Wilk and Levene’s tests, respectively. Due to non-normal data distribution, non-parametric methods were applied. Hence, the non-parametric statistical methods showed distinct group-level differences in the kidney damage indices. The Kruskal–Wallis test revealed significant differences across treatments (*p* < 0.001), and Mann–Whitney U tests identified specific pairwise differences. The degenerative and proliferative lesions were most prominent in fish exposed to 2,4-D at 100 µg/L (IK = 34), followed by pirimiphos-methyl and propamocarb hydrochloride. Inflammatory changes were mainly observed in the pirimiphos-methyl groups. The histopathological lesions were concentration-dependent, with 2,4-D causing irreversible renal damage at higher concentrations. These findings highlight the nephrotoxic risks posed by common pesticides and validate that the use of histopathological indices, combined with robust non-parametric testing, provides a reliable approach to evaluating organ-specific pesticide toxicity. These biomarkers offer sensitive early warning indicators of environmental risk, reinforcing the suitability of common carp as a model species for ecotoxicological assessment.

## 1. Introduction

Pesticides are chemicals that prevent, destroy, repel, or mitigate the presence of pests. They can also be used as plant regulators, defoliants, or desiccants [1]. The global use of pesticides, which poses a risk of exposure to all organisms and the environment, has increased significantly in recent years [2,3]. Therefore, the application of pesticides is recognised as the most considerable intentional input of biologically active substances into terrestrial ecosystems [4]. Over the past few decades, the intensification of international trade has been accompanied by a notable 50% increase in pesticide application, from 1990 to 2022. The rise in pesticide use has reflected its environmental and health implications, especially in low-income regions, where pesticide application has increased due to agricultural growth [5]. Thus, today, modern agriculture is highly dependent on the use of pesticides, primarily for crop protection and yield enhancement [6,7,8,9]. The benefit of their application is undeniable, but on the other hand, there is clear evidence that the widespread importation of pesticide substances causes irreversible damage to ecosystems and their inhabitants, including humans [7,10,11].

Fish are essential bioindicators for pollution in aquatic ecosystems, as they allow the use of biological approaches in environmental biomonitoring. The changes that occur in their bodies as a result of pollution can serve as reliable biomarkers for determining the degree and type of pollution in aquatic ecosystems. In ecotoxicological studies, biomarkers are valuable tools in monitoring the health of aquatic ecosystems. Incorporating complex biomarkers at different levels of biological organisation is a suitable approach to detect pollutant-induced responses [12]. Biomarkers are also essential assessment tools because they provide specific information about the biological effects of a particular pollutant. They can be used for monitoring purposes and to clarify the causal relationship between the risk to aquatic health and the concentration of the toxicant. In this regard, morphological methods are applied in ecotoxicological research because they provide the opportunity to assess the effects of pollutants on specific target organs and tissues. Additionally, morphological changes in cellular and tissue structure are crucial parameters in determining the potential toxicity of contaminants. By studying the extent of each morphological change, the sensitivity of an organism to the level of toxicity to which it is exposed can also be determined.

The kidneys of fish play a key role in maintaining body fluid homeostasis, as well as in the excretion of toxic xenobiotic residues. Studies related to pathological disorders in kidney tissues can also be considered an important parameter that indirectly reveals the intensity of environmental pollution [13]. Therefore, fish kidneys can be successfully applied in ecotoxicological studies; however, they are less commonly used compared to fish gills and liver. Their function and the established changes in the histological structure under the influence of various toxicants are associated with disruptions in homeostasis [14]. On this basis, conducting research related to morphological changes can provide information about the state of the studied organ as a result of a particular environmental pollutant’s action, which can affect the health of the whole organism [15].

The present laboratory study aims to assess, for the first time, the histopathological changes in the kidneys of common carp (*Cyprinus carpio* Linnaeus, 1758) after 96-h exposure to three pesticides (pirimiphos-methyl, propamocarb hydrochloride, and 2,4-dichlorophenoxyacetic acid) at their experimental concentrations. To our knowledge, this is the very first study on the toxicological effects of pirimiphos-methyl and propamocarb hydrochloride on fish renal health.

## 2. Materials and Methods

### 2.1. Experimental Fish

The common carp is one of the most preferred species for industrial breeding among freshwater fish species. It is widely distributed in almost all countries worldwide and is an essential fish for human consumption [16,17]. The common carp has a high commercial value worldwide. Still, it is also used as a bioindicator for water pollution [18] because it is a demersal fish with feeding habits that expose it to various types of environmental pollutants. In addition, it is easily caught and maintained under laboratory conditions [19].

### 2.2. Experimental Pesticides

Pirimiphos-methyl is an organophosphorus insecticide that is often used for the prevention and control of insects during the storage of agricultural production [20]. However, residues in the crop resulting from its excessive application pose a health hazard to humans and animals [21]. It is widely used in major grain-producing countries to protect against insect attack, as this organophosphate insecticide has a long-lasting activity [22]. Pirimiphos-methyl is also widely used against populations of the malaria mosquito *Anopheles* spp. (Meigen 1818), especially in Africa [23,24]. The mechanisms of resistance to pirimiphos-methyl are poorly understood; however, organophosphates, like carbamates, block the action of the acetylcholinesterase (AChE) enzyme by competitively binding to its active site [25]. Therefore, pirimiphos-methyl is responsible for the phosphorylation of AChE, which regulates the hydrolysis of acetylcholine in the synaptic cleft of the insect nervous system [26,27,28]. Just like AChE, the circulating enzyme butyrylcholinesterase (BchE), also known as pseudocholinesterase, is a target for phosphorylation and inhibition by this insecticide [29].

Propamocarb hydrochloride was first introduced into the European market in 1978 to control pathogens in ornamental crops and certain vegetables [30]. The fungicide exhibits good protective and curative activity against downy mildew, with no phytotoxic effects observed in fruits and vegetables, including tomatoes, potatoes, and cucumbers [31,32,33,34,35]. It belongs to the carbamate pesticide family and limits fungal growth by inhibiting the phosphoric acid and fatty acid synthesis pathways [36,37]. This pesticide is classified as slightly toxic by oral, dermal, and eye acute contact and practically nontoxic by inhalation [38]. Propamocarb hydrochloride has systemic activity after absorption through the leaves, stems, and roots and by transport through the vascular system in treated plants [39].

2,4-Dichlorophenoxyacetic acid (2,4-D) is a synthetic auxin herbicide that, due to its effectiveness, selectivity, low cost, and broad spectrum in pest control, has become one of the most frequently used herbicides in agricultural and urban areas worldwide [40]. It is commonly used to control dicotyledonous weeds in cereal crops, such as maize (*Zea mays* L.) and wheat (*Triticum aestivum* L.). Despite its agricultural use for over eight decades, the development of resistance to 2,4-D has been slow, with 25 weeds globally having developed resistance to 2,4-D as of 2021 [41]. In 2012, 2,4-D was the most widely used herbicide in home and garden areas, roughly equal to glyphosate used in combined non-agricultural regions [42]. Furthermore, 2,4-D is registered as an ingredient in approximately 1500 agricultural and domestic pesticides, either as the sole active ingredient or in combination with others [43,44]. However, even at low concentrations, 2,4-D has herbicidal effects on dicotyledons due to its ability to induce uncoordinated cell growth [45], damage to vascular tissues and roots, and malformation of leaves and stems [46]. Studies have shown that this herbicide can bioaccumulate in non-target organisms when exposed for a short period [47,48]. Due to its high water solubility, acidic characteristics, and mobility, 2,4-D has become a significant concern for soil and groundwater contamination [49,50], posing a serious environmental and health risk to non-target organisms [51,52].

### 2.3. Experimental Set-Up

Juvenile fish (*n* = 105, 6 months old) were used in the present experiment. They were purchased from the Institute of Fisheries and Aquaculture in Plovdiv, Bulgaria, where they are bred and raised under strictly controlled conditions. The experimental individuals were of the same size and age group (Table 1), and they did not exhibit any external pathological changes. For the experiment, 15 individuals were used for each experimental concentration, as well as in the control group.

Fish were acclimated under static conditions for one week prior to the experiment. Seven 100-L aquaria were used: six were filled with dechlorinated water containing different pesticide concentrations, and one served as the control with no added chemicals. Two sublethal concentrations were tested for each pesticide: pirimiphos-methyl (10 μg/L and 60 μg/L), propamocarb hydrochloride (40 μg/L and 80 μg/L), and 2,4-D (50 μg/L and 100 μg/L) (Table 2). The concentrations were selected based on LC_50_ values provided by the manufacturers—600,000 μg/L for pirimiphos-methyl (96 h, aquatic invertebrates), 80,000 μg/L for propamocarb hydrochloride, and 100,000 μg/L for 2,4-D—corresponding to 1/60,000 and 1/10,000 (pirimiphos-methyl), and 1/2000 and 1/1000 (propamocarb and 2,4-D) of their respective LC_50_ values. The greater dilution of pirimiphos-methyl reflects its significantly higher toxicity as an organophosphorus compound.

These exposure levels were chosen based on LC_50_-derived calculations and our previous laboratory trials with similar pesticides in aquatic toxicology, ensuring sublethal yet biologically relevant conditions for histopathological assessment. While the studied pesticides are not currently included in Directive 2013/39/EU and are not routinely monitored in surface waters, the selected concentrations are comparable to or slightly higher than those occasionally reported in agricultural runoff and contaminated aquatic systems. For instance, pirimiphos-methyl has been detected up to 45 μg/L, propamocarb up to 70 μg/L, and 2,4-D up to 120 μg/L in surface waters [20,21,30,31,40,51].

All aquariums were equipped with aerator pumps to supply the necessary oxygen levels. The fish were fed daily with pelleted dry food (Aller Aqua, Golub-Dobrzyń, Poland) at a rate of 3–5% of their body weight. Water parameters, including temperature (°C), pH, dissolved oxygen (mg/L), and electrical conductivity (μS/cm), were measured three times a day in all aquaria (Table 3) until the experiment was terminated after 96 h [53]. The experiments were repeated twice.

### 2.4. Histopathological Assessment

The fish were dissected according to the procedure described by Rosseland et al. (2003) [54], which complied with the requirements for the humane treatment of experimental animals as outlined in Directive 2010/63/EU [55]. The animal study protocol was approved by the Research Ethics Committee at Paisii Hilendarski University of Plovdiv, Faculty of Biology (protocol code No. 13/14.05.2025; date of approval: 14 May 2025). The histopathological processing of the materials was carried out according to the standard methodology of Romeis (1989) [56], which includes fixation of the sample in 10% neutral buffered formalin, tissue processing (alcohol dehydration, xylene clearing, paraffin wax infiltration), embedding, microtome sectioning (Leica RM 2125 RTS) and hematoxylin and eosin (H&E) staining. The prepared slides were evaluated using a Leica DM 2000 microscope and imaging software (LAS V4.13). The histopathological changes in the kidney were assessed using the scale described by Bernet et al. (1999) [57]. The alterations were classified into four groups: circulatory, proliferative, degenerative, and inflammation-related changes, with each group encompassing specific changes that affected functional units of the organ or the entire organ. In addition, a 5-point scale was used to determine the severity of each change according to Saraiva et al. (2015) [58], as follows: (0)—no changes in the kidney structure (up to 10% of the organ structure); (1)—very mild changes in the kidney structure (from 10% to 20% of the organ structure); (2)—mild degree of changes in the kidney structure (from 20% to 30% of the organ structure); (3)—moderate degree of changes in kidney structure (from 30% to 50% of the organ structure); (4)—severe degree of changes in the kidney structure (from 50% to 80% of organ structure); (5)—very severe degree of changes in the kidney structure (over 80% of the organ structure). The pathological degree of each change was determined using the significance factor (W) according to Bernet et al. (1999) [57]—W is a constant value and is categorised according to the author as follows: (1)—minimal pathological significance, reversible change after cessation of the toxicant; (2)—low pathological significance, the lesion is reversible in most cases if the stressor is neutralized; (3)—high pathological significance, irreversible changes, leading to partial or complete loss of organ function. The indices of histopathological changes are the result of the product of the degree of each specific change in the group multiplied by W. The summation of these final values for the organ determines the index (I) for the respective group of changes (circulatory I_C_, degenerative I_R_, proliferative I_P_, and inflammatory I_I_). The sum of all indices also determines the organ index (I_O_) for pathological change—I_O_ refers to a class, according to Zimmerli et al. (2007) [59], as follows: class I (Index ≤ 10)—normal histological structure with mild pathological changes (reversible); class II (Index 11–20)—normal histological structure with moderate pathological changes (reversible); class III (Index 21–30)—moderate degree of change in the histological structure (reversible); class IV (Index 31–40)—severe degree of change in the histological structure (irreversible); class V (Index > 40)—very severe degree of change in the histological structure (irreversible).

### 2.5. Statistical Analysis

Before statistical testing, the data were assessed for normality using the Shapiro–Wilk test and for homogeneity of variances using Levene’s test. As assumptions for parametric tests were not met, non-parametric methods were applied. The Kruskal–Wallis H test was used to evaluate the overall differences in the histopathological index values (I_k_) among the treatment groups [60]. When significant differences were detected, pairwise comparisons were conducted using the Mann–Whitney U test [61]. A significance threshold of *p* < 0.05 was used, and the pairwise differences were adjusted for multiple testing where appropriate. A bar plot was generated to display the final index for organ (I_k_) values per treatment, visualizing the group differences. Different lowercase letters were used to denote the statistically distinct groups based on the post hoc tests. Furthermore, a Principal Component Analysis (PCA) was performed on the kidney histopathological scores to evaluate the separation of treatment groups based on the histopathological alterations. The dataset comprised 22 histopathological parameters assessed across six experimental groups (*n* = 10 per group), including six pesticide-treated groups. The control group was excluded from the PCA, as all histopathological scores for the control specimens were zero, resulting in no variance and thus no contribution to the analysis. The experimental groups were as follows: (Pir-10), exposed to 10 µg/L pirimiphos-methyl; (Pir-60), exposed to 60 µg/L pirimiphos-methyl; (Pro-40), exposed to 40 µg/L propamocarb hydrochloride; (Pro-80), exposed to 80 µg/L propamocarb hydrochloride; and (2,4-D 50) and (2,4-D 100), exposed to 50 and 100 µg/L 2,4-dichlorophenoxyacetic acid, respectively. The following histopathological variables were used in the PCA: Kidney-level changes included haemorrhage (KHem), hyperaemia (KHyp), and aneurysms (KAne); Tubular degenerative changes consisted of vacuolar degeneration (TVacDeg), hyaline degeneration (THyalDeg), necrobiosis (TNecBio), and necrosis (TNec); Glomerular lesions included dilatation of Bowman’s capsule (GDilBow), contraction (GCont), necrobiosis (GNecBio), and necrosis (GNec); Interstitial tissue changes involved necrosis (INec); Proliferative responses were assessed through hypertrophy and hyperplasia in tubules (TProlHyp and TProlHpl), glomeruli (GProlHyp and GProlHpl), and thickening of Bowman’s capsular membrane (GBowThick); Interstitial tissue proliferation was evaluated through hypertrophy (IProlHyp) and edema (IEdema); Inflammatory reactions in the kidney were described by infiltration (KInfl) and activation of melano-macrophages (KActMM). All variables were standardised before analysis using z-score transformation to ensure comparability. PCA was performed using the scikit-learn library [62], and the first two principal components (PCA1 and PCA2) were visualized in a two-dimensional scatter plot. Each treatment group was represented with a unique colour and symbol. Confidence ellipses (95%) were added to highlight the distribution of individuals within each treatment group. Vectors representing histopathological parameters were overlaid to illustrate their contribution to the separation of treatments, except for variables located at or near the origin, which were excluded from the labelling to avoid visual clutter. The final figure was generated in Python using matplotlib [63] and seaborn for visual aesthetics. All statistical analyses were conducted using Python 3.10 with the scipy.stats and seaborn libraries.

## 3. Results and Discussion

**Figure 1:**
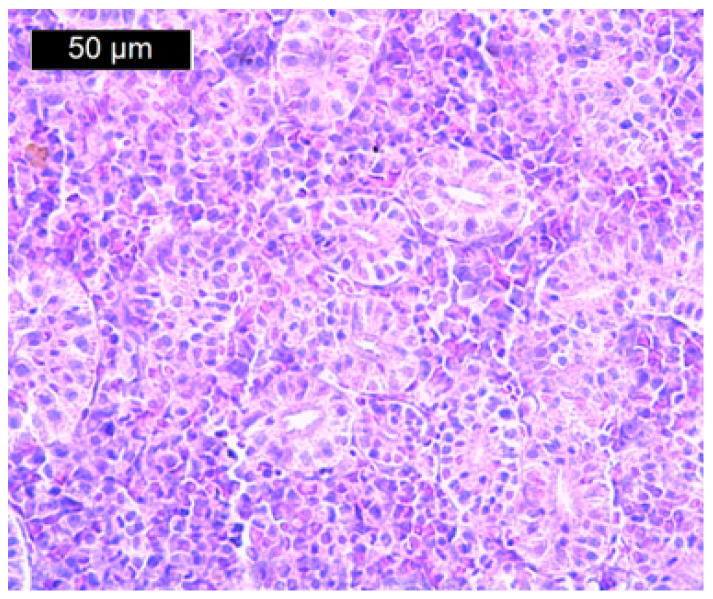
A normal histological structure of the kidney from the common carp control group ×400, H&E.

The results showed normal morphology of the histological structure of the kidney in the control fish group. According to the five-point (0–5) scale for the severity of changes, the histopathological sections of the kidney were assigned a grade of 0. However, in some individuals, the histopathological changes were found to occupy less than 10% of the section surface. The normal histological structure of a common carp kidney is shown in Table 4 and Figure 1.

The teleostean kidney consists of the anterior kidney (also known as the head kidney) and the posterior kidney (also known as the body or trunk kidney). Embryologically, the anterior kidney derives from the pronephros, and the posterior kidney from mesonephros [64]. The anterior kidney is integrated into the endocrine system of fish. It plays a crucial role in the stress response, mediated by the hypothalamic–pituitary–interrenal cell (HPI) axis and the hypothalamic–sympathetic nervous system–chromaffin tissue (HSC) axis [65,66]. In addition, the head of the kidney contains endocrine elements, including chromaffin cells and interrenal tissue, which are located around the blood vessels. The posterior kidney contains the nephrons with variable quantities of hemopoietic and lymphoid tissue in the interstitium. It is mainly composed of renal corpuscles that are made up of the Bowman’s capsules, glomeruli, renal tubules, and collecting ducts. In vertebrates, three types of kidneys are found: the pronephros, mesonephros, and metanephros [67,68]. Therefore, the healthy kidney is involved in the maintenance of body fluid homeostasis, it produces urine, which acts as an excretory route for the metabolites of a variety of xenobiotics to, which the fish may be exposed, it also excretes other nitrogen-containing waste products from metabolism, such as ammonia and creatinine and last but not least, the kidney performs an important function related to electrolyte and water balance and the maintenance of a stable internal environment [69].

The histopathological sections of the pirimiphos-methyl-treated fish kidney revealed no significant changes in the circulatory system, including haemorrhage, hyperemia, and aneurysms. Therefore, the index of these changes (I_KC_) at both tested concentrations was 0, respectively (Table 4).

**Figure 2:**
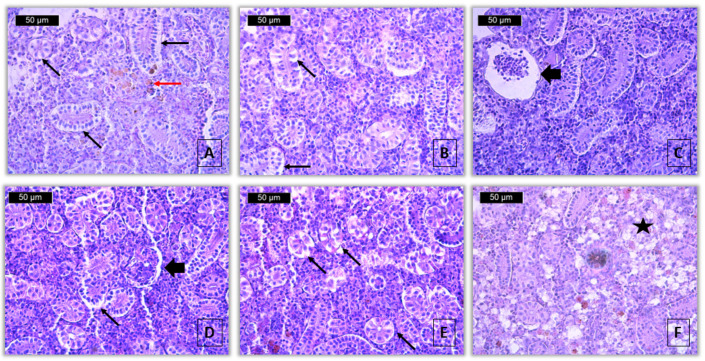
Histopathological alterations in kidney of common carp after acute (96-h) exposure with pirimiphos-methyl (H&E): (**A**)—activation of melanomacrophages (red arrow) and vacuolar degeneration (thin black arrow) (10 μg/L), ×400; (**B**)—vacuolar degeneration (thin black arrow) (10 μg/L), ×400; (**C**)—dilatation of Bowman’s capsule (thick black arrow) (10 μg/L), ×400; (**D**)—dilatation of Bowman’s capsule (thick black arrow) (60 μg/L), ×400; (**E**)—vacuolar degeneration (thin black arrow) (60 μg/L), ×400; (**F**)—hypertrophy of interstitial tissue (star) (60 μg/L), ×400.

Degenerative changes were observed in the renal parenchyma, localized in the renal tubules, glomeruli, and interstitial hematopoietic tissue. Considering the degree of involvement of the disorders, a general trend of increasing severity was observed with the increasing concentration of pirimiphos-methyl applied. In the cytoplasm of single epithelial cells of the covering epithelium, which lines the wall of the renal tubules, vacuolar degeneration with a mild degree of manifestation was detected, similarly at both tested concentrations (Figure 2A,B). Along with this, a more severe degenerative change was also observed in the epithelial cells, namely hyaline-droplet degeneration. In single cells, processes of necrobiosis are also observed, associated with changes in the nucleus, including karyopyknosis, karyorrhexis, and karyolysis. Necrosis was also reported in the epithelial cells of the renal tubules. The hyaline-droplet degeneration, necrobiosis, and necrosis were detected, but only at the higher applied concentration, and the degree of manifestation was determined as mild.

In the area of the Bowman’s capsule in the glomerulus, a well-pronounced dilatation was observed, which, with the involvement of the organ, was determined to a mild degree at a concentration of 10 μg/L and a moderate degree at a concentration of 60 μg/L pirimiphos-methyl (Table 4, Figure 2C,D). Shrinkage of the renal corpuscles was observed to a very mild degree, similar at both concentrations. Necrobiosis and necrosis of the glomerulus, as well as necrosis of the interstitial tissue, were not observed at any of the concentrations used. The degenerative change index (I_KR_) was 5 at a concentration of 10 μg/L and 12 at a concentration of 60 μg/L.

Proliferative changes were present in the renal tubules, glomeruli and interstitial tissue. Mild renal tubule hypertrophy was found at both tested concentrations, while tubular hyperplasia was not observed. Hypertrophy and hyperplasia of the glomerulus were also not detected. A thickening of the membrane of the Bowman’s capsule in the glomerulus was observed only at the higher concentration to a very mild degree. Among the changes in the interstitial tissue, moderate hypertrophy and very mild edema were observed at both concentrations tested (Figure 2E). The proliferative change index (I_KP_) at a concentration of 10 μg/L is 7, and at a concentration of 60 μg/L it is 9.

Regarding the changes related to inflammation, lymphocyte infiltration was not observed. Melanomacrophage activation was found to be moderate at both tested concentrations (Figure 2A). The index of these changes (I_KI_) was 6 for both concentrations.

After calculating the indices of all changes, it was found that the kidney index (I_K_) of fish treated with pirimiphos-methyl at a concentration of 10 μg/L was 18, and in those treated with a concentration of 60 μg/L, it was 27. Based on the results obtained and the scale proposed by Zimmerli et al. (2007) [59], the I_K_ at a concentration of 10 μg/L pirimiphos-methyl falls into class II (index 11–20), which means that the organ has a normal histological structure with moderate pathological changes that are reversible. Moreover, the I_K_ at a concentration of 60 μg/L pirimiphos-methyl falls into class III (index 21–30), which indicates that the organ has a moderate degree of change in the histological structure that is still reversible.

The histopathological examination of kidney sections from common carp exposed to pirimiphos-methyl for 96 h revealed no significant circulatory system alterations, such as haemorrhage, hyperemia, or aneurysms, at either of the tested concentrations (10 μg/L and 60 μg/L). Accordingly, the Index for Circulatory Changes (I_KC_) remained 0 across all groups. This was confirmed by statistical analysis, where the Kruskal–Wallis test showed no significant differences between treatment groups (Kruskal–Wallis test, *Hc* = 0, *p* = 1.000) (Table 4).

In contrast, degenerative changes were observed in the renal parenchyma, affecting the renal tubules, glomeruli, and interstitial hematopoietic tissue. The index for degenerative changes (I_KR_) showed a concentration-dependent increase (control: 0, 10 μg/L: 5, 60 μg/L: 12). Alterations included vacuolar degeneration (mild, at both concentrations), hyaline-droplet degeneration, necrobiosis (including karyopyknosis, karyorrhexis, and karyolysis), and necrosis, all observed at the higher concentration. These changes were visible in the cytoplasm and nuclei of single epithelial cells lining the renal tubules (Figure 2A,B). The statistical analysis showed significant differences between groups (Kruskal–Wallis test, *Hc* = 73.80, *p* < 0.001), and post hoc comparisons confirmed significant pairwise differences across all groups (Mann–Whitney U test, *p* < 0.001) (Table 4).

Proliferative changes were also observed, including tubular and interstitial hypertrophy, as well as mild edema. The index for proliferative changes (I_KP_) increased from 0 (control) to 7 (10 μg/L) and 9 (60 μg/L). These changes were statistically significant (Kruskal–Wallis test, *Hc* = 47.69, *p* < 0.001), with significant differences between the control group and both exposure groups (Mann–Whitney U test, *p* < 0.001), while the difference between the two exposed groups was not statistically significant (Mann–Whitney U test, *p* = 0.1335) (Table 4).

Regarding inflammatory processes, the primary finding was the activation of melanomacrophages in both exposed groups. The index for inflammatory changes (I_KI_) increased from 0 in the control group to 3 in the exposed group. The Kruskal–Wallis test indicated a significant difference among groups (Kruskal–Wallis test, *Hc* = 12.53, *p* < 0.01), with post hoc tests showing significant differences between the control and both treatment groups (Mann–Whitney U test, *p* < 0.01), but no significant difference between the two concentrations (Mann–Whitney U test, *p* = 0.5776) (Table 4).

The total organ index (I_K_) increased progressively with concentration: 0 (control), 18 (10 μg/L), and 27 (60 μg/L). Statistical analysis confirmed this trend with a highly significant difference between groups (Kruskal–Wallis test, *Hc* = 125.4, *p* < 0.001), and all pairwise comparisons were statistically significant (Mann–Whitney U test, *p* < 0.001) (Table 4).

The kidneys of fish treated with propamocarb hydrochloride revealed changes in the circulatory system of the organ in single areas in only one of the individuals. Since the changes do not cover the necessary commitment of the authority to be included in the scale of degree of changes (0–5), this change was assigned a degree of 0, no changes. Therefore, the index of these changes (I_KC_) was also 0 (Table 5).

**Figure 3:**
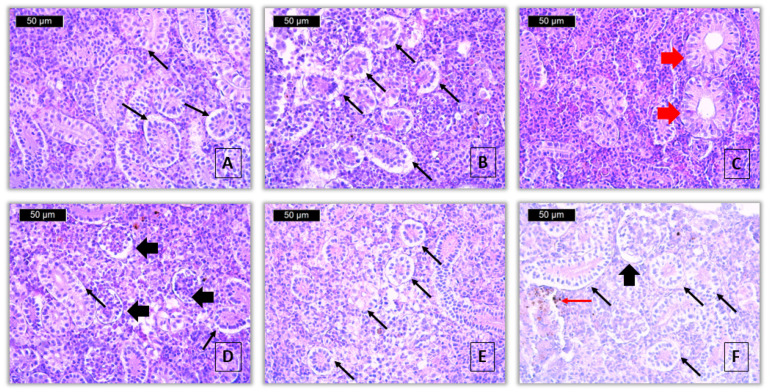
Histopathological alterations in kidney of common carp after acute (96-h) exposure with propamocarb hydrochloride (H&E): (**A**,**B**)—vacuolar degeneration (thin black arrow) (40 μg/L), ×400; (**C**)—hypertrophy of the tubule (thick red arrow) (40 μg/L), ×400; (**D**)—dilatation of Bowman’s capsule (thick black arrow) and vacuolar degeneration (thin black arrow) (80 μg/L), ×400; (**E**)—vacuolar degeneration (thin black arrow) (80 μg/L), ×400; (**F**)—vacuolar degeneration (thin black arrow), dilatation of Bowman’s capsule (thick black arrow) and activation of melanomacrophages (thin red arrow) (80 μg/L), ×400.

Vacuolar degeneration in the cytoplasm of the renal tubular epithelial cells was detected to a very mild extent at the lower concentration, while at the higher concentration, it was determined to be moderately pronounced (Figure 3A,B,D–F). Hyaline-droplet degeneration in the epithelial cells of the renal tubules was observed to a very mild extent at a concentration of 40 μg/L and to a mild extent at a concentration of 80 μg/L. Necrobiosis and necrosis of the renal tubules were not detected.

Dilatation of the Bowman’s capsule in the glomerulus was found and was expressed to a mild extent at both tested concentrations (Figure 3D,F). Shrinkage of the renal corpuscles, necrobiosis, and necrosis in the glomerulus, as well as necrosis in the interstitial hematopoietic tissue, were not observed at both tested concentrations of the pesticide. The index of degenerative changes (I_KR_) in the kidney of treated fish after exposure to propamocarb hydrochloride at the lower concentration was 4, while at the higher tested concentration, the value was 7.

Regarding the proliferative changes in the renal tubules, hypertrophy was observed (Figure 3C), affecting epithelial cells to a very mild degree, similar at both concentrations. At the same time, hyperplasia was not detected at any of the concentrations tested. Of the proliferative changes in the glomerulus, hypertrophy was reported to be to a very mild degree, similar at both concentrations. Additionally, at both experimental concentrations, hypertrophy was observed in the glomerulus, with a very mild degree of manifestation, while hyperplasia was absent in the analyzed histological sections. Thickening of the membrane of the Bowman’s capsule in the glomerulus was recorded only at the higher concentration of the applied pesticide, with a very mild degree of manifestation. Proliferative changes in the interstitial tissue were not detected at any of the fungicide concentrations. The calculated index of proliferative changes (I_KP_) in the kidney of fish treated with a concentration of 40 μg/L was 2, while in those treated with 80 μg/L fungicide, the value was 4.

Of the inflammatory changes in the kidney, lymphocyte infiltration was not observed at either of the fungicide concentrations tested. Melanomacrophage activation was detected to a very slight extent at a concentration of 40 μg/L, and a slight extent at a concentration of 80 μg/L (Figure 3E). The calculated inflammatory index (I_KI_) for the lower concentration was 2, while for the higher concentration, the value was 4. The total kidney index (I_K_) of fish treated with propamocarb hydrochloride at a concentration of 40 μg/L was 8, and at a concentration of 80 μg/L it was 15 (Table 5).

According to the scale proposed by Zimmerli et al. (2007) [59], the results obtained indicate that the IK at the lower concentration falls into class I (index ≤ 10), indicating that the organ has a normal histological structure with mild, reversible pathological changes. At the higher applied concentration, I_K_ falls into class II (index 11–20), which indicates that the kidney of the treated fish has a normal histological structure and the presence of moderate pathological changes, which are also reversible. The results suggest that the changes in the kidney increase in direct proportion to the rise in the applied concentrations of propamocarb hydrochloride.

The kidney of common carp exposed to propamocarb hydrochloride for 96 h showed no significant changes in the circulatory system (Kruskal–Wallis test, *Hc* = 0, *p* = 1.000). Therefore, all samples received a score of 0 for the circulatory system alterations, and the index for circulatory changes (I_KC_) was 0 in all groups (Table 5).

In contrast, degenerative changes were observed in the renal parenchyma, particularly within the tubular epithelium. Vacuolar degeneration was noted to a very mild extent (score 1) at 40 μg/L, and increased to a moderate degree (score 3) at 80 μg/L. Similarly, hyaline-droplet degeneration was scored as very mild (1) and mild (2) at the lower and higher concentrations, respectively. Necrobiosis and necrosis of the renal tubules were not observed at either concentration. In the glomeruli, dilatation of Bowman’s capsule was found at a mild level (score 2) in both exposed groups (Figure 3A,B,D–F). The index for degenerative changes (I_KR_) increased accordingly: 0 (control), 4 (40 μg/L), and 7 (80 μg/L). The statistical analysis showed a significant difference between the treatment groups (Kruskal–Wallis test, *Hc* = 34.04, *p* < 0.001). The pairwise comparisons confirmed significant differences between all group pairs (Mann–Whitney U test, *p* < 0.05) (Table 5).

Proliferative changes in the kidney were also evident. Tubular hypertrophy was observed at both concentrations with a very mild degree of expression (score 1), while hyperplasia was absent. In the glomeruli, hypertrophy was also scored as very mild at both exposure levels. Thickening of Bowman’s capsule membrane was noted only at the higher concentration, with a very mild degree of change. No proliferative changes were found in the interstitial tissue (Figure 3C). The index for proliferative changes (I_KP_) increased from 0 (control) to 2 (40 μg/L) and 4 (80 μg/L). These differences were statistically significant (Kruskal–Wallis test, *Hc* = 29.83, *p* < 0.001), with pairwise comparisons confirming significant differences between all groups (Mann–Whitney U test, *p* < 0.05) (Table 5).

Regarding inflammatory processes, lymphocyte infiltration was not observed. However, activation of melanomacrophages was recorded at both concentrations—very mild (score 1) at 40 μg/L and mild (score 2) at 80 μg/L (Figure 3E). The index for inflammatory changes (I_KI_) was 0 (control), 2 (40 μg/L), and 4 (80 μg/L). The Kruskal–Wallis test revealed a significant difference between groups (Kruskal–Wallis test, *Hc* = 10.81, *p* < 0.01), with post hoc comparisons showing that the treated groups were significantly different from the control (Mann–Whitney U test, *p* < 0.01), but not from each other (*p* = 0.3783) (Table 5).

The overall organ index (I_K_), reflecting total kidney damage, increased progressively with concentration: 0 (control), 8 (40 μg/L), and 15 (80 μg/L). This increase was statistically significant (Kruskal–Wallis test, *Hc* = 74.5, *p* < 0.001), and all pairwise comparisons revealed significant differences (Mann–Whitney U test, *p* < 0.05) (Table 5).

Based on the classification of Zimmerli et al. (2007) [59], the total organ index (I_K_) of 8 in fish exposed to 40 μg/L of propamocarb hydrochloride places them in Class I (I_K_ ≤ 10), indicating a normal histological structure with mild, reversible pathological changes. At 80 μg/L, an I_K_ of 15 corresponds to Class II (I_K_ 11–20), suggesting a normal histological structure with moderate pathological changes that remain reversible (Table 5).

After a short-term treatment with the herbicide 2,4-D, changes in the circulatory system of the kidney, similar to those observed with the other two pesticides, were not detected. Therefore, the index of these changes (I_KC_) at both applied concentrations was 0 (Table 6).

**Figure 4:**
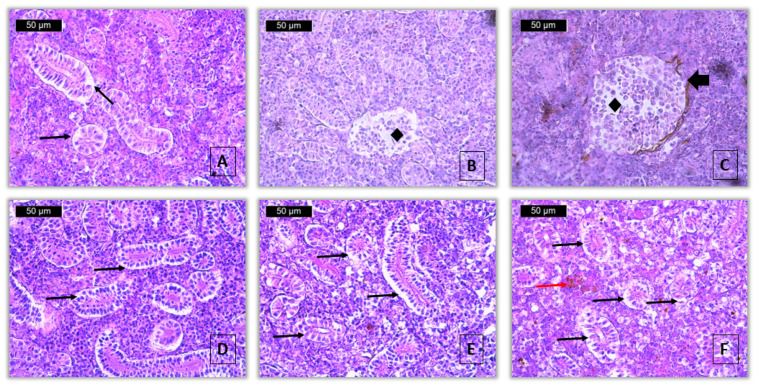
Histopathological alterations in kidney of common carp after acute (96-h) exposure with 2,4-D (H&E): (**A**)—vacuolar degeneration (thin black arrow) (50 μg/L), ×400; (**B**)—necrosis of glomerulus (rhombus) (50 μg/L), ×400; (**C**)—necrosis of glomerulus (rhombus) and dilatation of Bowman’s capsule (thick black arrow) (50 μg/L), ×400; (**D**,**E**)—vacuolar degeneration (thin black arrow) (100 μg/L), ×400; (**F**)—vacuolar degeneration (thin black arrow) and activation of melanomacrophages (thin red arrow) (100 μg/L), ×400.

Degenerative changes were reported in the epithelial cells of the renal tubules, expressed as vacuolar degeneration localized in the cytoplasm of the cells, which was determined to be mild at a concentration of 50 μg/L and severe at a concentration of 100 μg/L of 2,4-D (Figure 4A,D–F). Hyaline-droplet degeneration, necrobiosis, and necrosis were not observed at any of the applied concentrations. Of the degenerative changes in the glomerulus, dilation of the Bowman’s capsule was found to a very slight extent at the lower concentration and to a slight extent at the higher concentration of the applied pesticide (Figure 4C). Renal corpuscle shrinkage was observed to a very mild extent only at the higher pesticide concentration used. Necrobiosis, including karyorrhexis, karyopyknosis, and karyolysis, as well as glomerular necrosis, was observed to a very mild extent, similarly at both experimental concentrations (Figure 4B,C). The index of degenerative changes (I_KR_) in the kidney for the lower applied fungicide concentration is 5, while at the higher concentration it has a value of 15.

From the proliferative changes in the renal tubule, hypertrophy was found at a concentration of 100 μg/L to a very mild degree, while hypertrophy at a concentration of 50 μg/L, as well as hyperplasia, at both concentrations, were absent. Glomerular hypertrophy was described to be very mild at the lower concentration and moderate at the higher concentration. Glomerular hyperplasia was very mild, only at a concentration of 100 μg/L. Thickening of the membrane of the Bowman’s capsule in the glomerulus was not observed at any of the concentrations. Hypertrophy of the interstitial tissue was present to a very mild extent at both fungicide concentrations used. Edema in the interstitial hematopoietic tissue was found to be mild at a concentration of 50 μg/L, and severe at the higher concentration. The organ proliferative change index (I_KP_) at the lower concentration was 6, and at the higher concentration [15].

Among the changes associated with inflammatory processes, the activation of melanomacrophages was found, which was determined to be mild in both concentrations (Figure 4F). At the same time, lymphocytic infiltration was not detected in any of the used concentrations of 2,4-D. When calculating the inflammatory process index (I_KI_), a value of 4 was found for both fungicide concentrations.

The kidney index (I_K_) of fish treated with 2,4-D, at a concentration of 50 μg/L, was 15, while the index of those treated with a concentration of 100 μg/L was 34 (Table 6). Based on the results obtained and according to the scale proposed by Zimmerli et al. (2007) [59], it was calculated that the kidney index (I_K_) at the lower concentration falls into class II (index 11–20). This indicates that the organ has a normal histological structure with moderate, reversible pathological changes. However, at the higher concentration, I_K_ falls into class IV (index 31–40). This indicates a severe degree of change in the histological structure of the organ, and the processes are considered irreversible. The results suggest that the higher concentration of the applied fungicide 2,4-D causes significantly more serious pathological lesions in the histological structure of the fish kidney treated with this herbicide.

Following the 96-h exposure to the herbicide 2,4-D, no histopathological changes were observed in the circulatory system of the kidney, consistent with previous results obtained for pirimiphos-methyl and propamocarb hydrochloride. Consequently, the index for circulatory changes (IKC) was 0 in all treatment groups, and no statistically significant differences were found (Kruskal–Wallis test, *Hc* = 0, *p* = 1.000) (Table 6).

In contrast, degenerative changes were detected, primarily affecting the tubular and glomerular compartments. Vacuolar degeneration in renal tubular epithelial cells was determined to be mild at 50 μg/L and severe at 100 μg/L (Figure 4A,D–F). Hyaline-droplet degeneration, necrobiosis, and necrosis of the tubules were not observed at either concentration. In the glomeruli, dilatation of Bowman’s capsule was found at very mild (score 1) and mild (score 2) levels for the 50 and 100 μg/L groups, respectively (Figure 4C). Additional changes, such as glomerular contraction, necrobiosis (including karyopyknosis, karyorrhexis, and karyolysis), and necrosis, were observed to a very mild extent at both concentrations (Figure 4B,C). The index for degenerative changes (I_KR_) increased significantly with concentration: 0 (control), 5 (50 μg/L), and 15 (100 μg/L). These differences were statistically significant (Kruskal–Wallis test, *Hc* = 79.8, *p* < 0.001), with pairwise comparisons confirming significant differences between all groups (Mann–Whitney U test, *p* < 0.001) (Table 6).

Proliferative changes were also observed. Tubular hypertrophy was absent at 50 μg/L but present at 100 μg/L with a very mild degree (score 1). Glomerular hypertrophy was noted as very mild at 50 μg/L and moderate at 100 μg/L, while glomerular hyperplasia was observed to a very mild extent only at the higher concentration. Thickening of Bowman’s capsule was not present. In the interstitial tissue, hypertrophy was observed at both concentrations, and edema was detected as mild at 50 μg/L and severe at 100 μg/L. The Index for proliferative changes (I_KP_) rose from 0 (control) to 6 (50 μg/L) and 15 (100 μg/L). These increases were statistically significant (Kruskal–Wallis test, *Hc* = 70.49, *p* < 0.001), with all pairwise comparisons between groups showing significant differences (Mann–Whitney U test, *p* < 0.001) (Table 6).

Regarding inflammatory processes, lymphocytic infiltration was not observed at any concentration. However, activation of melanomacrophages was found to a mild extent in both exposed groups (Figure 4F). The index for inflammatory changes (I_KI_) was 0 (control) and 4 (for both 50 and 100 μg/L groups). These differences were statistically significant (Kruskal–Wallis test, *Hc* = 12.04, *p* < 0.01), though no significant difference was detected between the two exposure groups (Mann–Whitney U test, *p* > 0.05) (Table 6).

The total organ index (I_K_) showed a substantial dose-dependent increase: 0 (control), 15 (50 μg/L), and 34 (100 μg/L). These differences were highly significant (Kruskal–Wallis test, *Hc* = 152.7, *p* < 0.001), and all pairwise comparisons between groups were statistically significant (Mann–Whitney U test, *p* < 0.01) (Table 6).

Based on the classification system of Zimmerli et al. (2007) [59], the I_K_ score of 15 at 50 μg/L places the kidneys into Class II (index 11–20), indicating moderate pathological changes that are generally reversible. In contrast, the I_K_ score of 34 at 100 μg/L corresponds to Class IV (index 31–40), representing a severe and likely irreversible degree of histological alteration. These results demonstrate that the herbicide 2,4-D induces significantly more serious renal damage at higher concentrations in common carp (Table 6, Figure 4).

To assess the cumulative impact of each tested pesticide on kidney histopathology, the index for organ (I_K_) was calculated as the sum of severity scores across all observed alterations in each individual. This composite index allowed for the direct comparison of the overall kidney damage caused by different treatments.

The control group exhibited no histopathological changes, resulting in an I_K_ value of 0. Among the exposed groups, the lowest I_K_ value was recorded in fish treated with propamocarb hydrochloride at a concentration of 40 μg/L (I_K_ = 8), indicating a relatively low level of pathological damage. Pirimiphos-methyl at 10 μg/L and 2,4-D at 50 μg/L both resulted in moderate damage, with I_K_ values of 18 and 15, respectively. Slightly higher but still moderate alterations were observed in the kidneys of fish exposed to propamocarb hydrochloride at 80 μg/L (I_K_ = 15). Pirimiphos-methyl at 60 μg/L caused a more pronounced level of damage (I_K_ = 27). In comparison, the most severe histopathological response was observed following exposure to 2,4-D at 100 μg/L, with an I_K_ value of 34, representing substantial tissue disruption.

**Figure 5:**
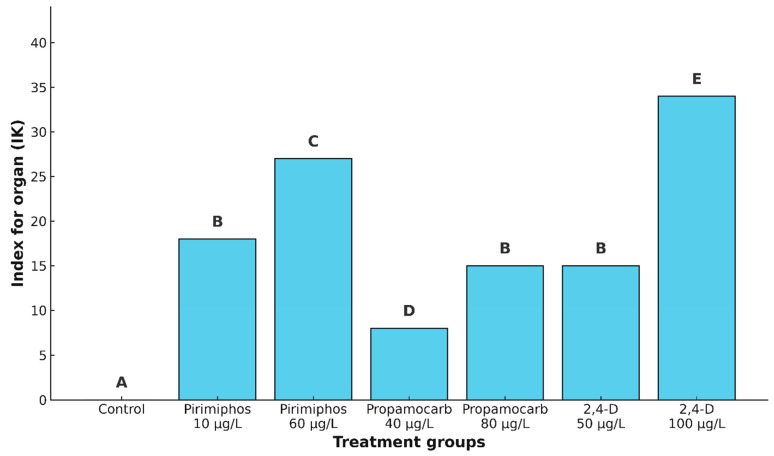
Index for organ (IK) in the kidney of common carp after acute (96-h) exposure to the tested concentrations of pirimiphos-methyl, propamocarb hydrochloride, and 2,4-D. Different letters indicate statistically significant differences between treatment groups (Mann–Whitney U test, *p* < 0.05).

The statistical analysis using the Kruskal–Wallis test revealed a highly significant difference in I_K_ values among the treatment groups (*Hc* = 58.70, *p* < 0.001). The pairwise post hoc analysis using the Mann–Whitney U test confirmed significant differences between most treatment pairs. Specifically, the control group differed significantly from all treated groups. However, no significant differences were found among the groups treated with pirimiphos-methyl at 10 μg/L, propamocarb hydrochloride at 80 μg/L, and 2,4-D at 50 μg/L (Mann–Whitney U test, *p* > 0.05). These treatments were therefore considered statistically similar in terms of their overall impact on kidney histopathology. In contrast, 2,4-D at 100 μg/L induced significantly more severe lesions than all other treatments, highlighting its strong nephrotoxic potential under the tested conditions (Figure 5).

**Figure 6:**
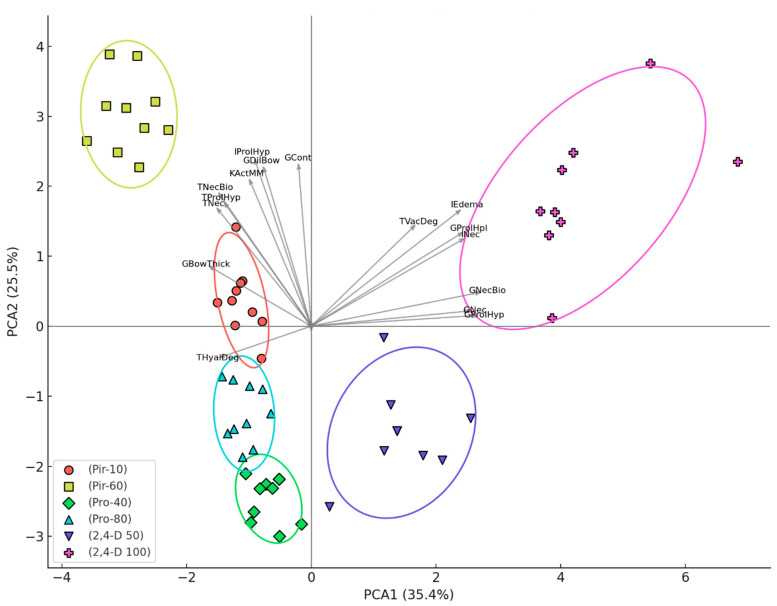
Principal component analysis (PCA) of kidney histopathological alterations in common carp following acute (96-h) pesticide exposure. PCA biplot showing the distribution of common carp (*n* = 10 per group) based on kidney histopathological alterations after 96-h exposure to different pesticide treatments. Each point represents an individual specimen, grouped by treatment and enclosed by 95% confidence ellipses. Vectors represent the histopathological variables contributing to separation along PCA1 and PCA2 axes (explaining 35.4% and 25.5% of the total variance, respectively); only vectors with meaningful influence (i.e., not located at the origin) are labelled. Treatment groups:(Pir-10): 10 µg/L pirimiphos-methyl; (Pir-60): 60 µg/L pirimiphos-methyl; (Pro-40): 40 µg/L propamocarb hydrochloride; (Pro-80): 80 µg/L propamocarb hydrochloride; (2,4-D 50): 50 µg/L 2,4-dichlorophenoxyacetic acid; (2,4-D 100): 100 µg/L 2,4-dichlorophenoxyacetic acid. Abbreviations of histopathological variables (vectors): Khem—haemorrhage; KHyp—hyperaemia; KAne—aneurysms; TVacDeg—vacuolar degeneration; THyalDeg—hyaline degeneration; TNecBio—tubular necrobiosis; TNec—tubular necrosis; GDilBow—dilatation of Bowman’s capsule; GCont—glomerular contraction; GNecBio—glomerular necrobiosis; GNec—glomerular necrosis; INec—interstitial necrosis; TProlHyp—tubular hypertrophy; TProlHpl—tubular hyperplasia; GProlHyp—glomerular hypertrophy; GProlHpl—glomerular hyperplasia; GBowThick—thickening of Bowman’s capsular membrane; IProlHyp—interstitial hypertrophy; IEdema—interstitial edema; KInfl—infiltration; KActMM—activation of melano-macrophages.

Figure 6 presents the principal component analysis (PCA) based on the histopathological scores from carp kidneys, showing a clear separation between treatment groups along the first two principal components (PCA1 = 35.4%, PCA2 = 22.5% of the total variance). A clear separation was observed among the treatment groups along the first two principal components. Notably, the group exposed to 100 µg/L 2,4-dichlorophenoxyacetic acid (2,4-D 100) formed a distinct cluster, primarily influenced by severe vacuolar degeneration of tubular cells, interstitial edema, glomerular hyperplasia, interstitial necrosis, glomerular necrobiosis, glomerular necrosis, and glomerular hypertrophy. In contrast, the groups treated with pirimiphos-methyl at both 10 µg/L (Pir-10) and 60 µg/L (Pir-60) were separated from others primarily due to thickening of Bowman’s capsular membrane, tubular necrosis, tubular hypertrophy, tubular necrobiosis, activation of melano-macrophages, interstitial hypertrophy, dilatation of Bowman’s capsule, and glomerular contraction. These patterns indicate that different pesticide compounds induced distinct histopathological responses, with glomerular and interstitial lesions being particularly influential in group differentiation. Vectors near the origin, representing negligible influence on separation, were intentionally left unlabeled for clarity. This multivariate approach reinforces the distinct pathological profiles induced by specific pesticide exposures (Figure 6).

Our findings are consistent with previously documented pesticide-induced histopathological effects in fish kidneys, including glomerular atrophy, vacuolization, hypertrophy, and necrosis [70,71,72]. However, our study is among the first to assess and statistically confirm the specific toxic profiles of pirimiphos-methyl and propamocarb hydrochloride under controlled laboratory conditions.

The results of the histopathological analysis of the kidney, following treatment with pirimiphos-methyl, propamocarb hydrochloride, and 2,4-D, revealed the presence of morphological changes in the organ’s structure. The observed proliferative and degenerative changes increased in direct proportion to the applied concentrations for all three experimental pesticides. Inflammatory changes in the organ were more pronounced compared to those in the liver, despite the lower degrees of manifestation of degenerative disorders. This was probably because the kidneys have a weaker regenerative potential than the liver. Circulatory changes were not observed in the kidneys after pesticide exposure.

Similar to our results on the toxic effects of pesticides on the normal histostructure of the fish kidney, many previous studies have reported that pesticides cause multiple histological changes in the kidney tissues of fish. Al-Otaibi et al. (2019) [70] demonstrated that diazinon exposure caused glomerular hypertrophy and haemorrhages in the kidney tissues of African sharptooth catfish (*Clarias gariepinus* Burchell, 1822). Inflammation and necrosis were observed in the kidney tissues of rainbow trout (*Oreochromis mykiss* Walbaum, 1792) exposed to the fungicide captan [73]. Additionally, Nile tilapia (*Oreochromis niloticus* Linnaeus, 1758) exposed to sumithione exhibited deformed and pyknotic nuclei, as well as vacuolization, in kidney tissues [74]. Cypermethrin caused necrosis, karyolysis, and ruptured renal tubules in Tengara catfish (*Mystus tengara* F. Hamilton, 1822) [75]. Cengiz et al. (2006) [76] and Vinodhini and Narayanan (2009) [77] also revealed similar changes in carp exposed to deltamethrin and toxic metals. The Java barb (*Barbonymus gonionotus* Bleeker, 1849) exposed to chronic sublethal concentrations of quinalphos exhibited renal changes, including degeneration of renal corpuscles, vacuolization, severely degenerated and dilated renal tubules, and hematopoietic tissue alterations, as well as changes in nuclear structure, mild to severe necrosis, and haemorrhage [78]. After glyphosate treatment, the renal tubules become severely dilated, and some of them are characterized by a loss of cellular integrity. Enlargement, edema, and hypertrophied nuclei are also observed. Glomeruli show vacuolization and disorganized blood capillaries. Necrosis and pyknotic nuclei are observed in the mesenchymal tissue [79]. Ayoola and Ajani (2008) [80] reported that exposure to cypermethrin caused tubular fusion and condensation of the glomerular contents. Ortiz et al. (2003) [81] reported epithelial lifting and necrosis in the tubule walls due to the toxic effects of lindane. Edema, atrophy of the tubule lumen, necrosis, pyknosis, congestion, and glomerular degeneration were found in the kidneys of fish treated with fipronil [71]. In fish exposed to deltamethrin for 24 h, the kidney showed degeneration in renal tubular epithelial cells, pyknotic nuclei in hematopoietic tissue, dilation of glomerular capillaries, narrowing of the tubular lumen, intracytoplasmic vacuoles in renal tubular epithelial cells with hypertrophied cells, and glomerular degeneration [69]. Dilatation of the tubular lumen diameter, pyknotic nuclei of some epithelial cells, hyaline degeneration, hydropic edema, and vacuolization were also observed in the renal tubules of common carp after exposure to dimethoate. Das and Mukherjee (2000) [82] reported dilatation of the renal tubules and necrotic changes characterised by karyorrhexis and karyolysis in rohu (*Labeo rohita* F. Hamilton, 1822) exposed to hexachlorocyclohexane. Tilak et al. (2001) [83] observed severe necrosis, edema in the renal tubules, cellular hypertrophy, granular cytoplasm, and vacuolization in the renal tissues of grass carp (*Ctenopharyngodon idella* Cuvier and Valenciennes, 1844) after exposure to fenvalerate. Degeneration in renal tubular epithelial cells, pyknotic nuclei in hematopoietic tissue, dilation of glomerular capillaries, glomerular degeneration, intracytoplasmic vacuoles in epithelial cells with hypertrophy and narrowing of the tubular lumen were observed in renal tissues of fish exposed to deltamethrin [69]. Velmurugan et al. (2007) [84] reported pyknotic nuclei in the renal tubular epithelium, hypertrophied tubular epithelial cells, glomerular shrinkage, and expansion of the space within the Bowman’s capsule in the kidney of mrigal (*Cirrhinus mrigala* Hamilton, 1822) exposed to monocrotophos. The result of histopathological examination revealed remarkable damage, such as vacuolization of the renal parenchyma and dilation of the renal tubules, in fish exposed to glyphosate [72]. Histopathological examination of kidney cells from African sharptooth catfish exposed to 2,4-D showed glomerular loss, vascular congestion, and intrarenal haemorrhage [85]. In contrast to these authors, at the same exposure but with a lower concentration of the herbicide in the glomerulus, our study found a low degree of dilation of the Bowman’s capsule and very mild necrobiotic changes in the epithelial cells of the renal tubules, as well as necrosis.

Overall, the observed histopathological alterations were grouped into four categories: changes in the circulatory system, proliferative changes, degenerative changes, and changes related to inflammatory processes. Post hoc Mann–Whitney U tests revealed specific pairwise distinctions, notably highlighting the severity of 2,4-D at 100 µg/L as the most toxic treatment, inducing irreversible alterations. The degenerative changes were more pronounced following exposure to 2,4-D and pirimiphos-methyl, with 2,4-D at 100 µg/L producing the highest index for degenerative changes (I_KR_ = 15). Proliferative responses also followed a concentration-dependent trend, with glomerular and interstitial hypertrophy being the most common. The inflammatory responses—particularly melanomacrophage activation were more intense in fish treated with pirimiphos-methyl, indicating potential immunological involvement.

## 4. Conclusions

In summary, we can conclude that this study confirms that pirimiphos-methyl, propamocarb hydrochloride, and 2,4-D induce dose-dependent histopathological alterations in the kidney of common carp, even during short-term (96-h) exposure. Along with this, compensatory-adaptive mechanisms were activated in the fish organ, which affected the kidney functionality and inevitably led to a deterioration in the overall fish health. There was a tendency towards an increase in morphological changes, and the degree of their expression is directly proportional to the increasing concentrations of the applied pesticides. The results of the present study on the identification of histopathological biomarkers in the bioindicator species common carp could be used to determine maximum permissible concentrations of organic pollutants in biota, as well as for ecological biomonitoring, applying an assessment model based on correlation dependencies between the identified biomarkers. The applied histopathological scoring system, reinforced by Kruskal–Wallis and Mann–Whitney U tests, effectively distinguished treatment effects and revealed significant concentration-dependent differences. These findings highlight the value of integrating morphological and statistical assessment to quantify sublethal organ damage in ecotoxicology. The study underscores the importance of regulating pesticide exposure levels and validates kidney biomarkers as effective tools for ecological risk assessment and environmental monitoring.

## Figures and Tables

**Table 1:** Length and weight of the experimental specimens.

Indicator	Control	Pirimiphos-Methyl		Propamocarb Hydrochloride		2,4-D	
		10 μg/L	60 μg/L	40 μg/L	80 μg/L	50 μg/L	100 μg/L
Total length (cm)	9.16 ± 0.4	10.44 ± 0.8	11.49 ± 0.5	9.64 ± 0.63	8.67 ± 2.9	9.31 ± 0.2	11.13 ± 0.3
Weight (g)	18.83 ± 5.7	19.25 ± 2.8	18.89 ± 2.4	19.11 ± 3.1	19.57 ± 2.7	16.28 ± 2.5	15.87 ± 3.5

**Table 2:** Experimental concentrations of the applied pesticides.

Pesticide	Recommended Dose	Dilution Relative to LC_50_	Experimental Concentration
Pirimiphos-methyl	0.00015 mL/m^3^–1 mL/dka	×60,000	10 μg/L
		×10,000	60 μg/L
Propamocarb hydrochloride	0.01–0.3 mL/dka	×2000	40 μg/L
		×1000	80 μg/L
2,4-D	120–200 mL/dka	×2000	50 μg/L
		×1000	100 μg/L

**Table 3:** Water parameters during the acute (96-h) pesticide exposure.

Pirimiphos-Methyl	pH	Temperature °C	Dissolved Oxygen mg/L	Electrical Conductivity μS/cm
Control	8.3 ± 0.05	17.5 ± 0.05	9 ± 0.05	365 ± 0.05
10 μg/L	8.2 ± 0.05	17 ± 0.05	8.5 ± 0.05	426 ± 0.05
60 μg/L	8.1 ± 0.05	17.5 ± 0.05	7.3 ± 0.05	473 ± 0.05
Propamocarb Hydrochloride	pH	Temperature °C	Dissolved Oxygen mg/L	Electrical Conductivity μS/cm
Control	8.5 ± 0.05	17.5 ± 0.05	9 ± 0.05	365 ± 0.05
40 μg/L	8.1 ± 0.05	18 ± 0.05	8.1 ± 0.05	433 ± 0.05
80 μg/L	7.9 ± 0.05	18.5 ± 0.05	7.5 ± 0.05	485 ± 0.05
2,4-D	pH	Temperature °C	Dissolved Oxygen mg/L	Electrical Conductivity μS/cm
Control	8.5 ± 0.05	17.5 ± 0.05	9 ± 0.05	365 ± 0.05
50 μg/L	7.5 ± 0.05	17.5 ± 0.05	8.2 ± 0.05	451 ± 0.05
100 μg/L	6.9 ± 0.05	18.5 ± 0.05	7.7 ± 0.05	469 ± 0.05

Legend: We measured the parameters three times per day, each day (*n* = 12).

**Table 4:** Histopathological changes in the kidney of common carp after acute (96-h) exposure to pirimiphos-methyl.

Reaction Pattern	Organ	Alteration	Importance Factor	Score Value		
				Control	Pirimiphos-Methyl	
					10 μg/L	60 μg/L
Changes in the circulatory system	Kidney	Haemorrhage	W_KC1_ = 1	0 ^A^	0 ^A^	0 ^A^
		Hyperaemia	W_KC2_ = 1	0 ^A^	0 ^A^	0 ^A^
		Aneurysms	W_KC3_ = 1	0 ^A^	0 ^A^	0 ^A^
Index for the circulatory system				I_KC_ = 0 ^A^	I_KC_ = 0 ^A^	I_KC_ = 0 ^A^
Degenerative changes	Tubule	Vacuolar degeneration	W_KR1_ = 1	0 ^A^	2 ^B^	2 ^B^
		Hyaline degeneration	W_KR2_ = 1	0 ^A^	0 ^A^	1 ^B^
		Necrobiosis	W_KR3_ = 2	0 ^A^	0 ^A^	1 ^B^
		Necrosis	W_KR4_ = 3	0 ^A^	0 ^A^	1 ^B^
	Glomerulus	Dilatation of the Bowman’s capsule	W_KR5_ = 1	0 ^A^	2 ^B^	3 ^C^
		Contraction	W_KR6_ = 1	0 ^A^	1 ^B^	1 ^B^
		Necrobiosis *	W_KR7_ = 2	0 ^A^	0 ^A^	0 ^A^
		Necrosis	W_KR8_ = 3	0 ^A^	0 ^A^	0 ^A^
	Interstitial tissue	Necrosis	W_KR9_ = 3	0 ^A^	0 ^A^	0 ^A^
Index for the degenerative changes				I_KR_ = 0 ^A^	I_KR_ = 5 ^B^	I_KR_ = 12 ^C^
Proliferative changes	Tubule	Hypertrophy	W_KP1_ = 1	0 ^A^	2 ^B^	2 ^B^
		Hyperplasia	W_KP2_ = 2	0 ^A^	0 ^A^	0 ^A^
	Glomerulus	Hypertrophy	W_KP3_ = 1	0 ^A^	0 ^A^	0 ^A^
		Hyperplasia	W_KP4_ = 2	0 ^A^	0 ^A^	0 ^A^
		Thickening of Bowman’s capsular membrane	W_KP5_ = 2	0 ^A^	0 ^A^	1 ^B^
	Interstitial tissue	Hypertrophy	W_KP6_ = 1	0 ^A^	3 ^B^	3 ^B^
		Edema	W_KP7_ = 2	0 ^A^	1 ^B^	1 ^B^
Index for the proliferative changes				I_KP_ = 0 ^A^	I_KP_ = 7 ^B^	I_KP_ = 9 ^B^
Inflammation	Kidney	Infiltration	W_KI1_ = 2	0 ^A^	0 ^A^	0 ^A^
		Activation of melanomacrophages	W_KI2_ = 2	0 ^A^	3 ^B^	3 ^B^
Index for the inflammatory processes				I_KI_ = 0 ^A^	I_KI_ =6 ^B^	I_KI_ =6 ^B^
Index for the organ				I_K_ = 0 ^A^	I_K_ = 18 ^B^	I_K_ = 27 ^C^

In addition, a 5-point scale was used to determine the severity of each change according to Saraiva et al. (2015) [58], as follows: (0)—no changes in the kidney structure (up to 10% of the organ structure); (1)—very mild changes (10–20% of the organ structure); (2)—mild changes (20–30%); (3)—moderate changes (30–50%); (4)—severe changes (50–80%); (5)—very severe changes (over 80%). Different superscript letters (^A^, ^B^, ^C^) indicate statistically significant differences between groups, as determined by the Mann–Whitney U test (*p* < 0.05). * Necrobiosis includes karyopyknosis, karyorrhexis, and karyolysis.

**Table 5:** Histopathological changes in the kidney of common carp after acute (96-h) exposure to propamocarb hydrochloride.

Reaction Pattern	Organ	Alteration	Importance Factor	Score Value		
				Control	Propamocarb Hydrochloride	
					40 μg/L	80 μg/L
Changes in the circulatory system	Kidney	Haemorrhage	W_KC1_ = 1	0 ^A^	0 ^A^	0 ^A^
		Hyperaemia	W_KC2_ = 1	0 ^A^	0 ^A^	0 ^A^
		Aneurysms	W_KC3_ = 1	0 ^A^	0 ^A^	0 ^A^
Index for changes in the circulatory system				I_KC_ = 0 ^A^	I_KC_ = 0 ^A^	I_KC_ = 0 ^A^
Degenerative changes	Tubule	Vacuolar degeneration	W_KR1_ = 1	0 ^A^	1 ^B^	3 ^C^
		Hyaline degeneration	W_KR2_ = 1	0 ^A^	1 ^B^	2 ^C^
		Necrobiosis	W_KR3_ = 2	0 ^A^	0 ^A^	0 ^A^
		Necrosis	W_KR4_ = 3	0 ^A^	0 ^A^	0 ^A^
	Glomerulus	Dilatation of the Bowman’s capsule	W_KR5_ = 1	0 ^A^	2 ^B^	2 ^B^
		Contraction	W_KR6_ = 1	0 ^A^	0 ^A^	0 ^A^
		Necrobiosis *	W_KR7_ = 2	0 ^A^	0 ^A^	0 ^A^
		Necrosis	W_KR8_ = 3	0 ^A^	0 ^A^	0 ^A^
	Interstitial tissue	Necrosis	W_KR9_ = 3	0 ^A^	0 ^A^	0 ^A^
Index for the degenerative changes				I_KR_ = 0 ^A^	I_KR_ = 4 ^B^	I_KR_ = 7 ^C^
Proliferative changes	Tubule	Hypertrophy	W_KP1_ = 1	0 ^A^	1 ^B^	1 ^B^
		Hyperplasia	W_KP2_ = 2	0 ^A^	0 ^A^	0 ^A^
	Glomerulus	Hypertrophy	W_KP3_ = 1	0 ^A^	1 ^B^	1 ^B^
		Hyperplasia	W_KP4_ = 2	0 ^A^	0 ^A^	0 ^A^
		Thickening of Bowman’s capsular membrane	W_KP5_ = 2	0 ^A^	0 ^B^	1 ^B^
	Interstitial tissue	Hypertrophy	W_KP6_ = 1	0 ^A^	0 ^A^	0 ^A^
		Edema	W_KP7_ = 2	0 ^A^	0 ^A^	0 ^A^
Index for the proliferative changes				I_KP_ = 0 ^A^	I_KP_ = 2 ^B^	I_KP_ = 4 ^C^
Inflammation	Kidney	Infiltration	W_KI1_ = 2	0 ^A^	0 ^A^	0 ^A^
		Activation of melano-macrophages	W_KI2_ = 2	0 ^A^	1 ^B^	2 ^C^
Index for inflammatory processes				I_KI_ = 0 ^A^	I_KI_ = 2 ^B^	I_KI_ = 4 ^B^
Index for organ I_K_				I_K_ = 0 ^A^	I_K_ = 8 ^B^	I_K_ = 15 ^C^

In addition, a 5-point scale was used to determine the severity of each change according to Saraiva et al. (2015) [58], as follows: (0)—no changes in the kidney structure (up to 10% of the organ structure); (1)—very mild changes (10–20% of the organ structure); (2)—mild changes (20–30%); (3)—moderate changes (30–50%); (4)—severe changes (50–80%); (5)—very severe changes (over 80%). Different superscript letters (^A^, ^B^, ^C^) indicate statistically significant differences between groups, as determined by the Mann–Whitney U test (*p* < 0.05). * Necrobiosis includes karyopyknosis, karyorrhexis, and karyolysis.

**Table 6:** Histopathological changes in the kidney of common carp after acute (96-h) exposure to 2,4-D.

Reaction Pattern	Organ	Alteration	Importance Factor	Score Value		
				Control	2,4-D	
					50 μg/L	100 μg/L
Changes in the circulatory system	Kidney	Haemorrhage	W_KC1_ = 1	0 ^A^	0 ^A^	0 ^A^
		Hyperaemia	W_KC2_ = 1	0 ^A^	0 ^A^	0 ^A^
		Aneurysms	W_KC3_ = 1	0 ^A^	0 ^A^	0 ^A^
Index for changes in the circulatory system				I_KC_ = 0 ^A^	I_KC_ = 0 ^A^	I_KC_ = 0 ^A^
Degenerative changes	Tubule	Vacuolar degeneration	W_KR1_ = 1	0 ^A^	2 ^B^	4 ^C^
		Hyaline degeneration	W_KR2_ = 1	0 ^A^	0 ^A^	0 ^A^
		Necrobiosis	W_KR3_ = 2	0 ^A^	0 ^A^	0 ^A^
		Necrosis	W_KR4_ = 3	0 ^A^	0 ^A^	0 ^A^
	Glomerulus	Dilatation of the Bowman’s capsule	W_KR5_ = 1	0 ^A^	1 ^B^	2 ^C^
		Contraction	W_KR6_ = 1	0 ^A^	0 ^A^	1 ^B^
		Necrobiosis *	W_KR7_ = 2	0 ^A^	1 ^B^	1 ^B^
		Necrosis	W_KR8_ = 3	0 ^A^	1 ^B^	1 ^B^
	Interstitial tissue	Necrosis	W_KR9_ = 3	0 ^A^	0 ^A^	1 ^B^
Index for the degenerative changes				I_KR_ = 0 ^A^	I_KR_ = 5 ^B^	I_KR_ = 15 ^C^
Proliferative changes	Tubule	Hypertrophy	W_KP1_ = 1	0 ^A^	0 ^A^	1 ^B^
		Hyperplasia	W_KP2_ = 2	0 ^A^	0 ^A^	0 ^A^
	Glomerulus	Hypertrophy	W_KP3_ = 1	0 ^A^	1 ^B^	3 ^C^
		Hyperplasia	W_KP4_ = 2	0 ^A^	0 ^A^	1 ^B^
		Thickening of Bowman’s capsular membrane	W_KP5_ = 2	0 ^A^	0 ^A^	0 ^A^
	Interstitial tissue	Hypertrophy	W_KP6_ = 1	0 ^A^	1 ^B^	1 ^B^
		Edema	W_KP7_ = 2	0 ^A^	2 ^B^	4 ^C^
Index for the proliferative changes				I_KP_ = 0 ^A^	I_KP_ = 6 ^B^	I_KP_ = 15 ^C^
Inflammation	Kidney	Infiltration	W_KI1_ = 2	0 ^A^	0 ^A^	0 ^A^
		Activation of melano-macrophages	W_KI2_ = 2	0 ^A^	2 ^B^	2 ^B^
Index for the inflammatory processes				I_KI_ = 0 ^A^	I_KI_ =4 ^B^	I_KI_ =4 ^B^
Index for organ I_K_				I_K_ = 0 ^A^	I_K_ = 15 ^B^	I_K_ = 34 ^C^

In addition, a 5-point scale was used to determine the severity of each change according to Saraiva et al. (2015) [58], as follows: (0)—no changes in the kidney structure (up to 10% of the organ structure); (1)—very mild changes (10–20% of the organ structure); (2)—mild changes (20–30%); (3)—moderate changes (30–50%); (4)—severe changes (50–80%); (5)—very severe changes (over 80%). Different superscript letters (^A^, ^B^, ^C^) indicate statistically significant differences between groups, as determined by the Mann–Whitney U test (*p* < 0.05). * Necrobiosis includes karyopyknosis, karyorrhexis, and karyolysis.

## Data Availability

The original contributions presented in this study are included in the article. Further inquiries can be directed to the corresponding author.
